# A Case of COVID-19 Induced Thrombotic Thrombocytopenic Purpura

**DOI:** 10.7759/cureus.16311

**Published:** 2021-07-11

**Authors:** Karthik Shankar, Deanna L Huffman, Chelsea Peterson, Muhammad Yasir, Robert Kaplan

**Affiliations:** 1 Internal Medicine , Allegheny Health Network, Pittsburgh, USA; 2 Internal Medicine, Allegheny Health Network, Pittsburgh, USA; 3 Hematology & Oncology, Allegheny Health Network, Pittsburgh, USA

**Keywords:** thrombotic microangiopathy (tma), covid-19, acquired ttp, caplacizumab, multimer, adamts-13, plasmic score, virchow’s triad, von willebrand factor, hypercoagulation

## Abstract

Thrombotic Thrombocytopenic Purpura (TTP) is a challenging thrombotic diathesis which requires prompt diagnosis and therapeutic intervention in order to avoid life-threatening consequences. There are two forms of TTP, congenital and acquired, with the acquired form constituting about 90% of cases. Both forms are associated with a deficiency of ADAMTS-13, a metalloproteinase enzyme responsible for cleaving ultra-large von Willebrand factor (uLvWF), preventing its pathologic accumulation. Within the last year, many of the diverse and serious effects of the COVID-19 virus have come to recognition, with some of the most dire consequences involving devastating vascular and hematologic complications. As with many viruses, it seems that the endothelium and the vasculature are often prime targets.

Here, we report a case of a 30 year old male who was diagnosed with TTP approximately one week after a positive COVID-19 test result. He responded appropriately to plasma exchange (PLEX), caplacizumab, and steroids.

We believe it is important to investigate a potential link between these two conditions, as TTP has significant morbidity and mortality risk if left unattended. We hope that our report will contribute to a better understanding of this potential link.

## Introduction

Thrombotic thrombocytopenic purpura (TTP) is a rare disease that results in uLvWF- platelet microthrombi formation within the microvasculature, ultimately leading to tissue ischemia and end-organ damage [1]. The presentation of this disease is often characterized by the pentad of fever, thrombocytopenia, hemolytic anemia, renal dysfunction, and neurologic dysfunction [1]. However, the full pentad is often not present in many patients [1].

TTP results from a deficiency in the enzyme referred to as ADAMTS-13 (a disintegrin and metalloproteinase with a thrombospondin type 1 motif member 13) [1]. This deficiency can occur congenitally via a gene mutation or in an acquired fashion, driven by immune-mediated destruction (classic/acquired TTP) [1]. In either instance, the circulating level of ADAMTS13 enzyme is significantly reduced, allowing for the formation of large numbers of ulVWF-platelet microthrombi [2]. This results in the consumption of platelets in the thrombotic process, and the subsequent mechanical destruction of red cells as they traverse through the occluded microvasculature [2].

The most common triggers for acquired TTP currently include seasonal viral infections, immunosuppressive agents, human immunodeficiency virus, anti-platelet agents and pregnancy [2]. Within the last 12 months, a new trigger is now being reported in the form of the COVID-19 virus. We report a case of COVID-19 induced TTP.

## Case presentation

A 30 year old obese Caucasian male with no significant past medical history and in good health presented to an outside hospital with a one day history of low back pain, left flank pain, and hematuria. His vital signs revealed absence of fever and a blood pressure elevation to 156/106 mmHg. Physical examination was relatively unrevealing aside from mild bilateral lower quadrant abdominal tenderness. He did not exhibit any altered mentation. Initial lab work at the outside hospital showed creatinine elevation to 1.51 mg/dL, hemoglobin of 13.7 g/dL, platelet count of 9 k/mcl, total bilirubin of 2.8 mg/dL, lactate dehydrogenase of 1375 U/L, and ferritin of 1068 mcg/L (Table 1). Urinalysis showed 3+ blood and significant proteinuria. Peripheral smear showed schistocytes. Reticulocyte count was normal. Prothrombin time/ International Normalized Ratio, activated partial thromboplastin time, and fibrinogen levels were all within normal limits. He was evaluated by both Hematology and Nephrology at the outside hospital and there was concern for thrombotic thrombocytopenic purpura. He was given 1 unit of platelets and 2 units of fresh frozen plasma and transferred to our facility for further evaluation. Of note, a COVID PCR test was performed at the outside hospital prior to transfer to our facility, and results were pending at the time of transfer. The test was performed due to a reported COVID exposure about one week prior to the patient’s presentation.

**Table 1:** Trend of Lab Values in our patient throughout his hospital course. The numbers in the top row correspond to the day of the hospital admission.

Hospital Course	1	2 (initiation of plasma exchange and prednisone)	3 (initiation of caplacizumab –yhdp)	4	5	6	7 (plasma exchange stopped)	8	9
Creatinine (mg/dL)	1.51	1.14	1.09	0.85	0.82	0.79	0.83	0.85	077
Hemoglobin (g/dL)	13.7	11.6	11.1	10.7	11.1	11.4	11.3	11.7	12.5
Platelets (k/mcl)	9	9	4	28	91	185	251	294	374
Lactate Dehydrogenase (U/L)	1068	1061	641	266	227	216	221	208	210
Haptoglobin mg/dL	<10	-	-	-	-	79	95	105	129
Total Bilirubin (mg/dL)	2.8	2.3	2.4	0.9	0.5	0.5	0.4	0.5	0.6
ADAMTS13 Activity (%)	3	-	-	-	-	-	-	-	54
ADAMTS13 Inhibitor (BU)	0.6	-	-	-	-	-	-	-	0.0

On arrival at our facility on December 12th, we calculated his Plasmic Score to be 7, as he met all the criteria (Table 2) [3]. Haptoglobin was ordered and returned low at <10 mg/dL. His ADAMTS13 activity level was 3% (normal range > 66%), and his ADAMTS13 inhibitor level was 0.60 BU (normal range < 0.50 BU) (Table 1). He was treated with plasma exchange (PLEX) for a total of 6 days with prednisone 1 mg/kg /day and concurrently with caplacizumab at a dose of 11 mg subcutaneously for 7 days. He had an appropriate response in his laboratory markers, achieving complete hematologic remission (Table 1). Just prior to discharge, we were informed that his COVID PCR test at the outside hospital on December 6th had returned positive. We retested the patient at our facility with a repeat COVID PCR test that also returned positive. He was discharged in stable condition with a steroid taper with the plan to complete 30 days of daily outpatient subcutaneous caplacizumab. Two days after discharge, his ADAMTS13 activity and inhibitor level were 54% and 0.0 BU, respectively. Two weeks later, his, ADAMTS13 activity was 66%.

**Table 2:** Plasmic Score

Component	Point
Platelet count < 30x10^9	1
Hemolysis (indirect bilirubin > 2mg/dL, reticulocyte count > 2.5% OR undetectable haptoglobin)	1
No active Cancer	1
No history of solid-organ or stem-cell transplant	1
MCV < 90fL	1
INR < 1.5	1
Creatinine <177 micromol/L	1

## Discussion

TTP is a thrombotic diathesis associated with significant acuity if undetected or untreated expeditiously. Mortality rates are reported to be around 90% [1]. If recognized and treated appropriately, the mortality rate drops to 10% [1]. The relapse rate is reported to be as high as approximately 50% in the first 30 days after PLEX is discontinued [1].

Acquired TTP is far more prevalent than congenital TTP, constituting about 90% of all TTP cases [4]. The common triggers, as mentioned in the introduction, include seasonal viral infections, antiplatelet agents, immunosuppressive agents, human immunodeficiency virus, and pregnancy. Recently, there has been an increasing number of reported cases in which COVID-19 infection seems to have been the inciting factor. We reviewed three other reported cases of COVID-19 induced-TTP similar to ours.

In a case reported by Albiol et al., a 57 year old German woman presented to the hospital with a dry cough, inability to smell, and a low grade fever [5]. She was diagnosed with COVID-19. Five days into her hospital course, her lab parameters changed dramatically, as she developed hemolytic anemia and severe thrombocytopenia, with low ADAMTS13 activity and high inhibitor levels (Table 3) [5]. She responded appropriately to therapeutic plasma exchange. [5]

Capecchi et al. reported another similar case of a 55 year old Italian female with previous history of TTP secondary to a bacterial pneumonia who presented with malaise, fatigue, chest discomfort, and dyspnea, eventually leading her to the emergency room where she was diagnosed with COVID-19 [6]. After three days, she suffered a dramatic drop in her platelet count from 184,000 to 14,000 k/mcl [6]. She also developed hemolytic anemia [6]. ADAMTS13 activity and inhibitor levels were checked and TTP was confirmed [6]. She achieved complete hematological response after 12 days of therapy with PLEX/steroids and caplacizumab (Table 3) [6].

Beauleiu et al. reported a case of a 70 year old Canadian male who tested positive for COVID-19 nineteen days prior to presenting to the hospital for confusion. He experienced an acute seizure requiring intubation. Investigation revealed an extremely low platelet count of 18 k/mcl, hemoglobin of 6 mg/dL, along with positive hemolytic indices (LDH, reticulocyte count, and haptoglobin). ADAMTS13 activity and inhibitor levels confirmed TTP. He responded appropriately to seven days of prednisone and plasma exchange (Table 3) [7].

**Table 3:** Comparison of our case to other recent reports of COVID Induced TTP

Case	Our case	Albiol et al. [5]	Capecchi et al. [6]	Beaulieu et al. [7]
Age (years)	30	57	55	70
Time from COVID diagnosis to TTP diagnosis	Unclear but approximately 1 week	6 days	3 days	19 days
Platelets (k/mcl) – at time of diagnosis/at time of discharge	9 / 374	22 /220	14 / 207	18 / normalized
Lactate Dehydrogenase (U/L) – at time of diagnosis/at time of discharge	1068 / 210	1451 / 218	18015 / 221	Elevated / normalized
Haptoglobin – at time of diagnosis/at time of discharge	<10 / 129	-	<10 / 146	< 10 / normalized
ADAMTS13 Activity (%) – at time of diagnosis/at time of discharge	3 / 54	2 / 89	<3 / 59	<10 / 43
ADAMTS13 Inhibitor (BU) – at time of diagnosis/at time of discharge	0.6 / 0.0	5.2 / 2.5	31 / <12	Weakly positive / < 0.5

In our review, one striking aspect was the age of our patient. Acquired TTP generally occurs after the age of 40 [1]. We found it interesting that acquired TTP was diagnosed at such a young age. Perhaps this speaks to this trigger resulting in a distinct TTP pathophysiology.

Another aspect of our review that we found interesting was the variability in time from diagnosis of COVID-19 to the diagnosis of TTP. In some cases, the diagnosis of TTP occurred almost immediately after the diagnosis of COVID-19. However, we also noticed a lag period of 19 days in one of the cases between the diagnosis of COVID-19 and the diagnosis of TTP [7]. This may reflect how the patient’s immune system responds to the viral infection.

Although the pathogenesis of COVID-19 induced-hypercoagulability is not yet adequately defined, there have been proposed mechanisms to explain this cause and effect relationship [8]. Singhania et al. details how COVID-19 has a complete effect on all three components of Virchow’s triad - endothelial injury, stasis, and hypercoagulable state [9]. To begin with, the endothelial insult occurs as the virus enters the endothelial cells directly via angiotensin-converting enzyme 2 (ACE-2) receptors [9]. A paper written by Varga et al. showed that COVID-19 viral elements were found inside endothelial cells, supporting the notion that the virus undertakes in direct invasion. This leads to subsequent release of multiple inflammatory mediators which induce endothelial injury [10]. Singhania et al. goes on to state that the stasis component of Virchow’s triad is attributable to the fact that COVID-19 has hospitalized and immobilized so many patients, making them more prone to thrombosis. Lastly, Singhania et. al described how COVID-19 infected patients have a variety of coagulation factor abnormalities including elevated von Willebrand factor (vWF), factor VIII, D-dimer, and fibrinogen [9].

It is not entirely clear how viral infections can trigger acquired TTP. However, given the multiple severe responses the COVID-19 can elicit from both the immune system and the coagulation cascade, some reasonable conclusions can be drawn as to the pathogenesis behind COVID-19 triggered TTP. The innate immune system generates a powerful inflammatory response to the COVID-19 virus, with the release of a storm of mediators. One such potential conclusion is that COVID-19 can overwhelm the regulatory ability of ADAMTS-13 enzyme activity by driving the production of the ADAMTS-13 targets, namely vWF and uLvWF, into overdrive. This was the case in a recently published paper by Doevelaar et al. In a study of 75 patients with COVID-19 infection, vWF, ADAMTS13 and vWF multimer formation were analyzed [11]. It was found that COVID-19 infection was associated with almost a five-fold increase in vWF levels [11]. This supports the claim that COVID-19 infection can result in an explosive increase in circulating vWF (including uLvWF), which the body’s ADAMTS-13 enzyme activity cannot adequately regulate, resulting in an excess of unchecked uLvWF, diffuse microthrombi, and systemic ischemia.

## Conclusions

TTP is a diagnosis which requires prompt detection and intervention in order to make an appreciable impact on clinical outcomes. With a high mortality rate which can be rapidly reduced with timely diagnosis and treatment, it is imperative to be able to recognize all potential triggers of acquired TTP to avoid missing cases which, heretofore, would have gone unnoticed. We hope that our report will contribute to increasing awareness amongst clinicians and help to unravel the many complexities of the disease, thereby assisting future clinicians in achieving the best possible outcomes for all patients.
